# Returning personalized, genetic health test results to individuals of African descent or ancestry in precision medicine research

**DOI:** 10.1093/haschl/qxad066

**Published:** 2023-12-12

**Authors:** Rachele M Hendricks-Sturrup, Nora Emmott, Maryam Nafie, Lauren Edgar, Tracey Johnson-Glover, Kurt D Christensen, Stephanie Argetsinger, Christine Y Lu

**Affiliations:** Duke-Robert J. Margolis, MD, Center for Health Policy, Washington, DC 20004, United States; Duke-Robert J. Margolis, MD, Center for Health Policy, Washington, DC 20004, United States; Duke-Robert J. Margolis, MD, Center for Health Policy, Washington, DC 20004, United States; Southern Nevada Black Nurses Association, Las Vegas, NV 89127, United States; Southern Nevada Black Nurses Association, Las Vegas, NV 89127, United States; Department of Population Medicine, Harvard Pilgrim Health Care Institute and Harvard Medical School, Boston, MA 02215, United States; Department of Population Medicine, Harvard Pilgrim Health Care Institute and Harvard Medical School, Boston, MA 02215, United States; Department of Population Medicine, Harvard Pilgrim Health Care Institute and Harvard Medical School, Boston, MA 02215, United States

**Keywords:** precision medicine, genetic research, return of results, health equity, health policy

## Abstract

Today, many epidemiological studies and biobanks are offering to disclose individual genetic results to their participants, including the National Institutes of Health's All of Us Research Program. Returning hereditary disease risks and pharmacogenetic test results to study participants from racial/ethnic groups that are historically underrepresented in biomedical research poses specific challenges to those participants and the health system writ large. For example, individuals of African descent are underrepresented in research about drug–gene interactions and have a relatively higher proportion of variants of unknown significance, affecting their ability to take clinical action following return of results. In this brief report, we summarize studies published to date concerning the perspectives and/or attitudes of African Americans engaged in genetic research programs to anticipate factors in disclosure protocols that would minimize risks and maximize benefits. A thematic analysis of studies identified (*n* = 6) lends to themes centered on motivations to engage or disengage in the return of results and integrating research and care. Actionable strategies determined in reaction to these themes center on ensuring adequate system and health education support for participants and personalizing the process for participants engaging in return of results. Overall, we offer these themes and actionable strategies as early guidance to research programs, and provide recommendations to policy makers focused on fair and equitable return of genetic research results to underrepresented research participants.

## Introduction

An increasing number of epidemiological studies and biobanks are offering to disclose individual genetic results to their participants.^1-4^ While such efforts satisfy ethical mandates to respect the needs and interest of participants, the impact of returning genetic research results in large-scale population-based studies, where participants are often unselected for disease and participant–investigator relationships may be remote, is not well understood.^5^ Data on the real-world impact of the return of genomic research results among racial/ethnic groups that are historically underrepresented in biomedical research are especially limited. These knowledge gaps compound concerns that, without a thoughtful approach to implementation, the return of genomic research results could exacerbate existing disparities in genomic medicine.^6^

Addressing the needs of participants of African descent/ancestry or heritage in the United States (ie, African Americans), in particular, is critical for both scientific and social reasons. Genetics has frequently been used to stigmatize and marginalize the individuals identified (either by self or society) as Black populations, making them wary about genomics research and precision medicine.^7-9^ In addition, genetic testing typically provides less utility for individuals of African descent/ancestry, providing molecular explanations for disease less often, perhaps partially due to health system factors that may include, but not be limited to, low clinician electronic health record engagement for historically marginalized groups and thus lesser amounts of phenotype data that are required to accurately interpret the genetic basis of disease among such groups.^10^ Moreover, as data on the influence of pharmacogenetic variants in individuals of African descent/ancestry emerge, it is unclear whether pharmacogenetic research reports provided to such individuals are consistent across genetic research programs to help address health disparities.

Considering these issues, it is critical to offer early, evidence-based guidance to research programs seeking to develop or contemplate policies focused on the fair and equitable return of genetic research results. In this brief report, we provide such an offering through a review of studies published to date about the perspectives and/or attitudes of African Americans engaged in the return of genetic research results to anticipate factors in disclosure protocols that would minimize risks and maximize benefits.

## Data and methods

### Literature review

Two authors (N.E. and M.N.) conducted a literature search in the National Institute of Health's (NIH's) PubMed database from November through December 2022 using the following search terms: African American, African, Black, and “return of results.” Original articles published at any time reporting perspectives, attitudes, and/or experiences among individuals of African descent/ancestry with return of genetic results were assessed for inclusion in the review. We extracted the following information from included articles: study population, purpose, approach, key findings, and authors’ policy and practice recommendations regarding return of results. Given the dearth of studies on return of research results with a specific focus on Black individuals of African descent/ancestry, we sought studies that examined disclosure of both clinical and research-grade genetic findings.

### Thematic analysis

Two authors (R.M.H.-S. and K.C.) conducted a thematic analysis of literature review results and their respective authors’ recommendations using deductive data coding (to identify and develop emerging categorical themes and actionable strategies) in Microsoft Word and Excel (Microsoft Corporation), using constant comparative analysis in accordance with the grounded theory approach.^11^ The resulting codes were built into categorical and actionable themes that were deliberated upon and discussed among all members of the research team until strong (>95%) agreement was reached.

## Results

### Literature review

We identified only 6 studies that met our inclusion criteria and are indexed to date in PubMed, all of which were published between the years 2012 and 2021 and focused on return of research vs clinical results (see Table 1). Three studies in our scoping review were from a single study in 2012 involving a total of 45 participants. Across all studies, the number (*n*) of participants of African ancestry/descent ranged from 41 to 48. Four of the 6 studies were conducted between 2012 and 2014 and 2 were conducted in 2021, prior to the All of Us program’s return of results announcement in December 2022. Five of the 6 studies were qualitative, and only 1 study focused on return of results in adult vs pediatric populations of African descent/ancestry. Only 1 study enrolled a racial/ethnic subset of participants from another study where return of genetic results would occur.^16^

**Table 1.** Summary of key findings and recommendations across studies reporting Black or African-American participant attitudes and perspectives around return of genetic results.

Author (year)	Study population, purpose, and approach	Key findings and recommendations
Halverson and Ross (2012)^12^	*Population:* African-American parents (*n* = 45) of children receiving medical care from 2 facilities in Chicago, IL *Purpose:* Assess perspectives of parents regarding biobank participation and return of results for themselves and their children *Approach*: Pre- and post-engagement surveys and focus groups	After discussing the design of biobanks to allow/not allow return of results, most participants agreed that they would enroll themselves and their children. Participants advocated for return of their results. Opinions about the return of their own results vs results of their children were similar.
Halverson and Ross (2012)^13^	*Population:* Same as above *Purpose:* Assess changes in attitudes about biobank research, governance, and the return of results before and after deliberative engagement *Approach:* Pre- and post-engagement surveys and focus groups	Engagement did not change attitudes. Most participants expressed moderate to strong concern regarding genetic information privacy in research. Most participants from both health care facilities expressed interest in receiving actionable results. Participants from the federally qualified health center opposed return of non-actionable results, while participants from the university-based practice were more interested. Researchers need to consider the diversity within African-American populations when developing return-of-results protocols.
Halverson and Ross (2012)^14^	*Population:* Same as above *Purpose:* Evaluate therapeutic misconceptions of participants about participating in biobank-based research with the potential for return of results *Approach:* Focus groups conducted during 4 participant engagement sessions	Several participants believed that they could receive clinical care by participating in biobank research. Return of results plans may contribute to perceptions that biobank participation provides clinical utility.
Yu et al (2013)^15^	*Population:* African-American (*n* = 41) and non–African-American (*n* = 35) adults in Seattle/King County, WA *Purpose:* Evaluate attitudes regarding return of results from exome and whole-genome sequencing *Approach:* Focus groups	African-American participants were less willing to participate themselves or to allow their children to participate in hypothetical studies of genomic sequencing than non–African-American participants. African Americans were more likely than non–African-American adults to discuss (1) how results may inform management of current, rather than future, health; (2) concerns with law enforcement access to their identifiable genetic information; (3) the psychological impacts of accessing their results; (4) lack of access to health care, which might hinder their ability to follow up on actionable results; (5) the importance of faith and church communities in decision making; (6) the potential for people to believe they are sick based on test results; (7) desire for control of one's own results; and (8) benefits to future generations.
Lewis et al (2021)^16^	*Population:* African-American participants of the ClinSeq project (*n* = 49) *Purpose:* Assess participant perspectives on optimal engagement strategies and priorities for return of results *Approach:* Focus groups	Participants had clear preferences for certain types of results (eg, life-threatening results) over others (eg, ancestry-related results). Development of return of results protocols would benefit from community engagement that includes the following: (1) large numbers of participants to provide a diversity of opinions, (2) longitudinal engagement, and (3) reciprocal flow of information between researchers and participants.
West et al (2022)^17^	*Population:* Professional, personal, family, and/or community stakeholders (*n* = 76, 48 of whom are African American) *Purpose:* Assess stakeholders’ perspectives on returning nonactionable apolipoprotein L1 (*APOL1*) genetic results to African-American research participants *Approach:* In-depth interviews	Most interviewees supported the return of *APOL1* results. Few interviewees presenting professional perspectives expressed concern for potentially harming individuals or limited benefits. Emergent themes of benefits included (1) personal value, (2) expectations of future actionability, (3) respect and reciprocity, and (4) community/scientific benefit. Participants also perceived risks that included psychological burdens.

### Thematic analysis

Emerging categorical and actionable themes across these studies that may inform the development and implementation of earnest and equitable policy solutions and support are listed in Table 2. Emerging categorical themes centered largely on motivations to engage or disengage in return of results and integrating research and care. Themes captured as actionable strategies moving forward centered largely on ensuring adequate system and health education support for participants and personalizing the process for participants engaging in return of results.

**Table 2.** Emerging categorical themes and actionable strategies: returning genetic test results to individuals of African descent/ancestry.

Emerging categorical themes	
*Motivations to engage or disengage* Participants hold general interest in engaging in the return of results process. Engagement in return of results likely depends on the clinical nature or actionability of the results being conveyed. Adults may equally engage themselves and their children in return of results.	*Integrating research and care* The return of genetic research results may contribute to misconceptions around clinical utility of the studies, especially if return of results occurs in usual care (vs research) settings. Lack of access to follow-up health care may hinder participants’ ability to address actionable genetic testing results.
**Actionable strategies**	
*Support the system and educate* Provide system support to address psychological impacts or burdens following return of results. Discuss community, generational, and/or scientific benefits and other implications with participants. Create and implement system guidelines and policies that support educated decision making among participants.	*Personalize the process* Discuss religious and/or spiritual beliefs that exist around disease manifestation. Address participants’ concerns about and desires for genetic information privacy and control of test results. Demonstrate respect for and reciprocity with participants.

## Discussion

Very few studies have been published and indexed in PubMed to date about the return of genetic research results to African-American individuals as an underrepresented population. Our work highlights specific challenges facing large-scale population-based studies, including but not limited to the National Institute of Health's (NIH’s) All of Us Research Program, that are enrolling large numbers of participants from minoritized populations and returning individual genomic research results.^18-20^ Developing protocols and policies that are evidence-based and sensitive to the needs and preferences of groups whose perspectives and health genetic data are underrepresented in the scientific literature (ie, African Americans) will be challenging without further research and engagement that could build on the present themes and actionable strategies presented.

All of Us is a high-profile example of population-based research that is pioneering the return of genomic research results and facing these challenges. All of Us has been returning results derived from genetic analyses as part of its commitment to core values that include giving participants access to their information.^18-20^ All of Us entered a new phase at the end of 2022 when it started returning personalized health reports that contain information about genetic variants that may increase risks for disease (hereditary disease risks) and genetic variants associated with drug/medication metabolism (pharmacogenetic results).^18^ Because over 75% of All of Us participants to date are from populations that are traditionally underrepresented in biomedical research, protocols for the return of hereditary disease risks and pharmacogenetic results will need to be sensitive to the special opportunities and challenges of disclosing genomic research results to members of these populations.^18-20^ For example, the All of Us Public Data Browser does not stratify survey data about social determinants of health by race/ethnicity.^21^ The All of Us program should address these query limitations, as they can create barriers to communities being able to understand factors that may disproportionally affect marginalized populations’ ability to appreciate the diversity of the community.

Our findings do highlight a number of both common themes and instructive points that may improve the likelihood that return of genetic results programs in population-based studies, including but not limited to programs like All of Us, will improve outcomes in African-American participants. First and foremost, return of genetic research results is desired by African Americans not only for themselves but to also benefit their children and community as a whole. There are also expectations that African Americans will be engaged in a bidirectional process when protocols are developed, including decisions about the types of information that would be disclosed and withheld. Articles also emphasized how the communication of information needs to appreciate the diversity of perspectives within the African-American community, and how decision making about study participation and post-disclosure follow-up is often made after consultation with trusted others, including spiritual leaders.

Our work also identifies potential misunderstandings that research programs will need to address. Therapeutic misperceptions that engagement in programs like All of Us will provide clinical care need to be proactively addressed, including given the potential for participants to be informed about important genetic information that participants will need to manage themselves. Frame effects, where messages are framed according to individuals’ attitudes and behaviors, might drive misconceptions around the intent of research participation. Protocol designs need to consider and avoid such misconceptions, particularly for the return of results in usual care settings, where the line between research and care may be blurred.^22^ While these challenges are not unique to African Americans, they are likely to be more problematic for the community. For example, genetic testing typically identifies more variants of uncertain significance,^23^ and the importance of pharmacogenetic variants in individuals of African descent/ancestry (vs others) is poorly understood, raising questions about whether they accurately identify drug–gene interactions.^24^ In addition, even when genetic conditions are identified in patients of African descent, they may be at risk of receiving care that is inferior to the care received by patients of European descent because of systemic biases that presently exist in health care settings.^10,25,26^ Materials that proactively address these complications should be disseminated through NIH-supported communication routes to help members of the African-American community fully understand the intent and processes for the return of results. The All of Us program may benefit African Americans by engaging in the development of community-driven solutions that address challenges. A return of results process that educates and supports participants in their attempts to navigate subsequent and necessary follow-up screening and care may be particularly beneficial. Such efforts are likely to be appreciated, and may improve the recruitment and sustained involvement of study participants. not just in the African-American community but from many underrepresented subgroups facing similar challenges.

Importantly, the gene list that research programs consider for return of results potentially has important implications for health inequities. Diagnostic genetic testing often provides molecular explanations for disease less often in individuals of African descent, and early evidence suggests that the yield from genetic-screening programs will be lower for groups such as African Americans than for other groups.^27,28^ Selection of genes associated with conditions that disproportionately affect African Americans may help mitigate these disparities. For example, a specific protein change (Val142Ile) in the tetrameric thyroxine transport protein transthyretin (*TTR*) gene is implicated in hereditary transthyretin-related amyloidosis (hATTR), a potentially fatal autosomal dominant disorder that disproportionately affects individuals of African descent/ancestry.^29^*TTR* corresponds to the use of tafamidis (ie, Vyndaqel, manufactured by Pfizer) as a pharmacogenetic biomarker with a US Food and Drug Administration clinical pharmacology and clinical study labeling section, and is listed in the American College of Medical Genetics and Genetics’ (ACMG) current recommendations (v3.2) for reporting secondary findings in clinical exome and genome sequencing.^30-32^ Programs such as All of Us that plan to screen genes and return results based on earlier versions of curated lists (eg, v2.0 of the ACMG's recommendations for secondary findings disclosure) would omit *TTR*/hATTR.^33^ Expanding gene lists to address current recommendations for secondary findings disclosure, including *TTR*/hATTR, may help ensure a more equitable distribution of benefits from return of results.

Last, questions remain regarding whether programs like All of Us should provide participants with personalized reports containing details about genetic variants of unknown or undefined significance (VUS).^34^ Well-meaning policies that discourage the disclosure of VUS to minimize the risk of returning false-positive results could disproportionally minimize the potential benefits of return of results. This is especially true for participants of African descent/ancestry, given that they are more likely to receive VUS findings during diagnostic testing. Therefore, if and/or whenever information relating to VUS is provided to individuals of African descent/ancestry, it will be important to (1) develop and implement processes and policies that appropriately convey new information about genetic health risk through timely reanalysis of variants of unknown significance and (2) ensure that research participants and their children have pathways within the health system to receive any necessary health education and receive necessary follow-up screening and care upon receiving such results.

There are some limitations to our literature review. First, our literature search was conducted in only 1 database (PubMed). Second, the studies in our review relied exclusively on hypothetical research scenarios (vs actual experiences) to examine issues related to return of genetic research results to individuals of African descent/ancestry and included relatively small numbers of participants. These factors limit the generalizability of our findings and potential utility of our recommendations. Nonetheless, our findings are helpful to stimulate further research, engagement, and discussions involving patients/study participants, researchers, clinicians, policy makers, and other health care system stakeholders who face the challenges noted herein with the hopeful mission to leverage precision medicine research and care to reduce racial/ethnic health disparities.

## Conclusion

To date, limited studies report perspectives about return of genetic results to individuals of African descent/ancestry. Nonetheless, stakeholders engaged in the advancement of health equity and precision medicine should consider these thematic findings to address local- and system-level challenges that accompany returning genetic testing results to members of distinct racial/ethnic subgroups, including individuals of African descent/ancestry. More research is needed to validate and/or enrich themes highlighted herein noting privacy concerns, actionability of results, and psychological impacts/burdens upon receiving information about their genetic health risks and pharmacogenetic treatment implications/considerations. Our recommended strategies along these themes may help ensure, as a starting point, that participants and their children are supported in their attempts to learn about their genetic status, including VUSs, and navigate follow-up care upon return of clinically actionable results through supportive study protocols and policies.

## Supplementary Material

qxad066_Supplementary_Data
